# Features and treatment of surviving casualties in the Kunshan 'August 2' Explosion Accident: 40 case reports and literature review

**DOI:** 10.1186/cc14389

**Published:** 2015-03-16

**Authors:** J Liu, WU Wu

**Affiliations:** 1 Suzhou Municipal Hospital, Nanjing Medical University, Suzhou, China

## Introduction

The aim was to analyze the injury features and treatment strategies of surviving casualties in the explosion accident on 2 August 2014 in Kunshan city (Kunshan 'August 2' Explosion Accident).

## Methods

We retrospectively studied 40 cases of surviving casualties in the Kunshan 'August 2' Explosion Accident, and systemically reviewed the relevant literature.

## Results

A total of 40 cases were admitted to our hospital within 6.3 ± 0.8 hours after the explosion accident on 2 August 2014 in Kunshan city, including 28 males and 12 females. The major injury types included burn injury, inhalation injury, blast injury (lung, eye, eardrum, and so forth), traumatic brain injury and bone fractures. All cases suffered from burn injury caused by the explosion. The mean burned area in the surviving casualties accounted for 92 ± 14% total body surface area (TBSA) and those with third-degree burns for 77 ± 19% TBSA. Additionally, incidence rate of inhalation injuries was 97.5%. There were 34 cases complicated by multiple organ dysfunction syndrome, which accounted for 85.0%. The common organ dysfunction of surviving casualties included the lungs, circulation, liver, gastrointestinal tract, kidney, brain, and coagulation. During hospitalization, the most common infectious site in surviving casualties was a burn wound, followed by blood and lung. The most common infectious strain of bacteria was Gram-negative bacteria, which accounted for 91.3%. Further analysis showed that *Proteus mirabilis *ranked first in occurrence, followed by *Acinetobacter baumannii *and *Pesudomonas aeruginosa*, and *Klebsiella pneumonia *and *Enterobacter cloacae *ranked fourth and fifth. After intensive treatment, the mean 28-day mortality was 20.0% and 90-day mortality was 62.5%, mainly due to septic shock and multiple organ dysfunction syndrome.

## Conclusion

During the Kunshan 'August 2' Explosion Accident, burn injury was the leading cause of injuries. Most surviving casualties are accompanied by multiple organ dysfunction syndrome and infection.

